# Understanding the Cu-Zn brass alloys using a short-range-order cluster model: significance of specific compositions of industrial alloys

**DOI:** 10.1038/srep07065

**Published:** 2014-11-17

**Authors:** H. L. Hong, Q. Wang, C. Dong, Peter K. Liaw

**Affiliations:** 1 Key Laboratory of Materials Modification (Dalian University of Technology), Ministry of Education, Dalian 116024, China; 2 Mechanical Engineering Department, Sanming University, Sanming 365004, China; 3 Dept. of Materials Science and Eng., The University of Tennessee, Knoxville, Tennessee 37996-2200, USA

## Abstract

Metallic alloys show complex chemistries that are not yet understood so far. It has been widely accepted that behind the composition selection lies a short-range-order mechanism for solid solutions. The present paper addresses this fundamental question by examining the face-centered-cubic Cu-Zn α-brasses. A new structural approach, the cluster-plus-glue-atom model, is introduced, which suits specifically for the description of short-range-order structures in disordered systems. Two types of formulas are pointed out, [Zn-Cu_12_]Zn_1~6_ and [Zn-Cu_12_](Zn,Cu)_6_, which explain the α-brasses listed in the American Society for Testing and Materials (ASTM) specifications. In these formulas, the bracketed parts represent the 1^st^-neighbor cluster, and each cluster is matched with one to six 2^nd^-neighbor Zn atoms or with six mixed (Zn,Cu) atoms. Such a cluster-based formulism describes the 1^st^- and 2^nd^-neighbor local atomic units where the solute and solvent interactions are ideally satisfied. The Cu-Ni industrial alloys are also explained, thus proving the universality of the cluster-formula approach in understanding the alloy selections. The revelation of the composition formulas for the Cu-(Zn,Ni) industrial alloys points to the common existence of simple composition rules behind seemingly complex chemistries of industrial alloys, thus offering a fundamental and practical method towards composition interpretations of all kinds of alloys.

Solid-solution-based industrial alloys generally fall within narrow composition ranges but the specific rule according to which these compositions are selected is largely unknown. The alloys in standard specifications are basically developed via extensive trial-and-error efforts. Usually compositions and properties are not directly correlated because of the involvement of multi-scaled structures. Industrial alloys often undergo complicated fabrication processes, such as solidification and thermomechanical treatments. Each processing step brings in new structure variations to the alloys. Therefore, despite the obvious fact that industrial alloys are classified into different specifications, the alloy composition selection rule is far from being understood.

It is noticed that the industrial alloy fabrication processes generally include a high-temperature solution-treatment step, and the final structures are issued from a single-phase parent state. It is the stability of these parent phases that determines the room-temperature structures and eventually the performances. The best-known example is steels, which are generally related to the austenite state, and different steel types, such as austenite, martensite, ferrite, pearlite, etc., are produced out of different austenite stabilities. The structure of these parent states, being single-phase solid solutions, is characterized by short-range chemical orders. In this sense, the fabrication processes are so adopted as to bring in proper structural variations on the basic parent solid solutions, such as structural defects of different scales and phase transitions. The alloy design can be much simplified, as only a single-phase state is involved, and the composition can be directly related to the parent phase stability.

However, the structural description of solid solutions is problematic. So far solid solutions are at best expressed by statistical short-range-order parameters like the Warren-Cowley *α_n_* parameter^1^, due to the presence of disorders. This *α_n_* parameter reflects the site occupancy for the *n*^th^ shell of neighbors in a binary AB alloy, defined as 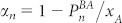, where 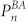 is the probability of finding an A atom in the neighborhood of a B atom, and *x_A_* and *x_B_* are respectively the proportions of atoms A and B in the alloy, with *x_A_*+ *x_B_* = 1. Though the heterogeneous distribution of solutes is well favored^2^, there has been no model for solid solutions that identifies the structural units on which possible composition rules rely, because a composition formula exists only when an averaged unit is present.

## Cluster-plus-glue-atom Model

We have attempted to unveil the structural units in Fe-containing Cu-Ni alloys^3^, maraging steels^4^, and β-Ti alloys^5^, etc., following a new structural approach, called the cluster-plus-glue-atom model, originally developed by us for quasicrystals and amorphous alloys^6^. In this model, any structure is described by a short-range structural unit consisting of a 1^st^-neighbor coordination polyhedral cluster and some glue atoms situated outside the clusters, expressed by a cluster formula [cluster]glue*_x_*. For a bulk metallic glass, the cluster is taken from a relevant devitrification phase, and the number of glue atoms is either 1 or 3. It was further pointed out that the total number of valence electrons per unit cluster formula for a bulk metallic glass is universally about 24^7^ so that the cluster formula for a bulk metallic glass resembles the ‘molecular’ unit of a chemical substance. The atomic structure of a bulk metallic glass is then viewed as a spatial arrangement of the 1^st^-neighbor cluster in a dense manner, and the 2^nd^-neighbor glue atoms fill the space between the clusters. The clusters are all isolated from each other in metallic glasses and quasicrystals, which is necessary to avoid the center-shell type of nearest-neighbor short-range orders to develop into longer-range ones.

Solid-solution alloys, being characterized by chemical short-range orders, would be treated in a similar manner. That is to say, there might be specific formulas that describe the chemical short-range-order local units in solid-solution structures. In the present research, as our first attempt toward understanding the general composition rule of industrial alloys, we will establish the cluster-plus-glue-atom model for the face-centered-cubic (FCC) solid solutions by examining the compositions of Cu-Zn α-brass industrial alloys. The Cu-Zn alloys are selected for the absence of any solid-state transition and for the large solubility of Zn in Cu that allows abundant alloy selections. The Cu-Zn system also represents solid solutions formed with solutes of negative enthalpies of mixing.

## Short-range Order in Cu-Zn Brasses

Cu-Zn brasses, like many industrial alloys, are based on solid solutions of a base metal, here the FCC Cu. In the equilibrium phase diagram^8^, the Cu-Zn solid solution covers a wide composition range, approaching 38.95 weight percent (wt.%) Zn at a high temperature. In the normal casting fabrication, Cu-Zn alloys exhibit a single α-phase FCC state below 35 wt.% Zn; above this Zn content, the intermetallic β-CuZn (CsCl type) would be formed, which induces precipitation strengthening, but at the expense of the reduced plasticity. For this reason, industrial Cu-Zn alloys contain at most about 40.0 wt.% Zn, which is slightly above the solubility limit of Zn in Cu.

Although Zn can be dissolved in the FCC Cu over a wide composition range, single-phase α-brass industrial alloys^9^ are located at specific compositions only, typically C21000 (gilding metal, 95Cu-5Zn, the number before the elements indicating wt.%), C22000 (commercial bronze, 90Cu-10Zn), C23000 (red brass, 85Cu-15Zn), C24000 (low brass, 80Cu-20Zn), C26000 (cartridge brass, 70Cu-30Zn), and C27000 (yellow brass, 65Cu-35Zn). It is noticed that many properties show obvious dependences on the Zn contents (for instance, see the property-composition graphs on page 296, American Society for Metals (ASM) Handbook^9^). Specifically, tensile strength rises rapidly with increasing the Zn content, showing an efficient solute-strengthening effect, and the rising tendency slows down above about 20 wt.% Zn. In accompany to the strength variations, the elongation first drops downwards and rises after 10 wt.% Zn.

**Figure 1:**
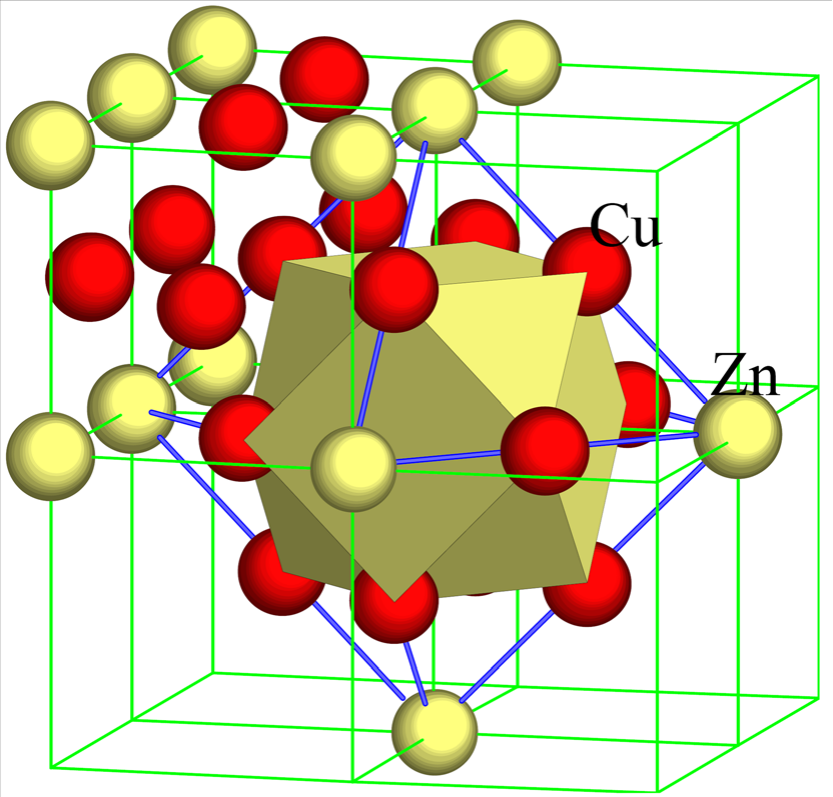
Structure of a Cu_3_Zn ordered state. The 1^st^ and 2^nd^ nearest-neighbor configurations of a possible low-temperature ordered Cu_3_Zn state with the AuCu_3_-structure type, where the twelve 1^st^ neighbors are occupied by Cu and the six 2^nd^ neighbors by Zn.

It has been long suspected that behind the many “anomalous” behaviors at specific Zn concentrations lies a short-range-order mechanism in α-brasses (see for instance^10^,^11^ and the references quoted therein), involving internal friction, stress relaxation, yielding, work-hardening, activation energy of creep, activity coefficient, specific heat, cold-working, electrical resistance, etc. The first direct evidence of short-range ordering was provided by a neutron-diffuse-scattering experiment in combination with a Monte Carlo simulation on an α-brass single crystal containing 31.1 atomic percent (at.%) Zn^12^. The Warren-Cowley short-range-order parameter for the nearest-neighbor position (1,1,0), α_1_ = −0.1373, is negative, signifying that the dissimilar Cu-Zn nearest order is favored. The α parameter for the second-nearest neighbor position (2,0,0) is positive, α_2_ = 0.1490, suggesting that the second neighbors are preferentially occupied by the Zn atoms. In accordance with this picture, the short-range order would reach eventually an ordered Cu_3_Zn state with the AuCu_3_-structure type. Figure 1 presents the 1^st^-neighbor cuboctahedral polyhedron [Zn-Cu_12_] and the 2^nd^-neighbor octahedron consisting of six Zn atoms, identified in Cu_3_Zn. A calculation of ground-state properties based on a Green's function technique^13^ confirmed that the mixing energies between Cu and Zn are always negative, ΔH_Cu-Zn_ < −6 KJ/mol, and, coincidently, the 1^st^-neighbor Warren-Cowley short-range-order parameter, α_1_, is always negative over the complete concentration range.

## Structural Model and Cluster Formulas of Solid Solutions for FCC α-brasses

**Figure 2:**
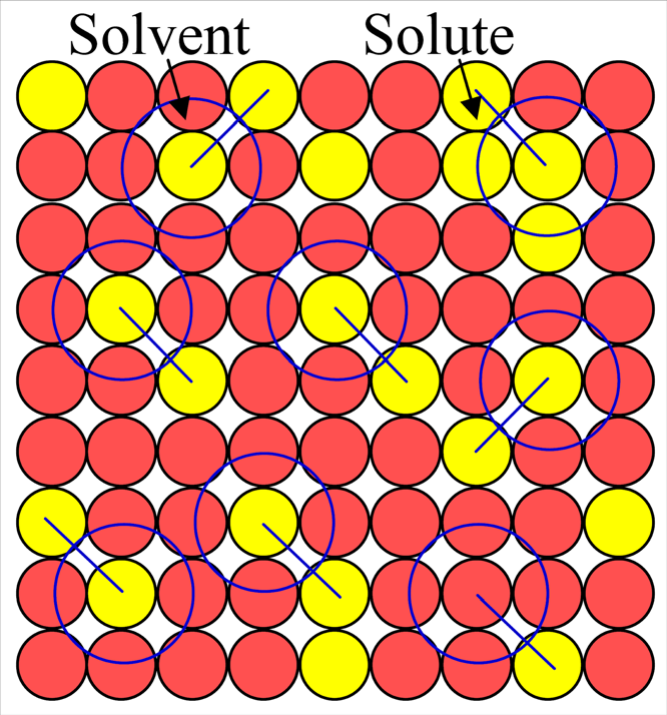
A two-dimensional solid-solution structure. Schematic diagram showing the distribution of solute atoms (yellow circles) in a square lattice of solvent atoms (light red circles). The major part of this structure can be represented by a local structural unit, formulated as [solute_1_-solvent_4_]solute_1_, which covers only the 1^st^-neighbors [solute_1_-solvent_4_] cluster (marked with a large blue circle) and one 2^nd^-neighbor solute as the glue atom (linked to the cluster center by a blue line segment).

Due to the difficulty in describing short-range orders, the relationship between the composition and the relevant short-range-order feature is not known. For the objective of extracting a simple formulism for the short-range orders in solid solutions, we here analyze a schematic two-dimensional solution structure shown in Figure 2, where solutes (yellow circles) are distributed in a solvent square lattice (light red circles), with a dissimilar inter-atomic bonding tendency between them. The major part of the structure is characterized by a local structural unit formulated with [solute_1_-solvent_4_]solute_1_, despite the presence of different local varieties, such as the solvent-richer down-right corner and the solute-richer upper-right corner in Figure 2. This formula covers only a 1^st^-neighbor cluster and some 2^nd^-neighbor glue atoms. This idealized local atomic configuration, complying with the inter-atomic interaction requirements, should show a relatively high structural stability against structures of nearby compositions and possibly possess specific properties in relevance to this local short-range order feature. Such a description might underline that the solid-solution alloys, though compositionally continuous, show special comprehensive properties at specific compositions where idealized short-range orders dominate. Hence, this cluster-based short-range-order structural model describes special ‘stable solid solutions’.

Thereof, we propose the following structural model for stable solid solutions to describe the ideal solute distribution in FCC α-brasses:

1. In accordance with the relatively - large negative Warren-Cowley short-range-order parameters for the 1^st^ neighbor (e.g., α_1_ = −0.1373 in 31.1 at.% Zn^12^), a Zn solute atom is nearest-neighbored by twelve Cu solvent atoms occupying the (1,1,0) × 2/*a* positions (*a* being the FCC lattice constant), forming a Zn-centered cuboctahedral cluster, [Zn-Cu_12_].
2. In accordance with the relatively - large positive Warren-Cowley short-range-order parameters for the 2^nd^ neighbor (e.g., α_200_ = 0.1490 in 31.1 at.% Zn^12^), the central solute Zn is 2^nd^-neighbored with Zn situating at the (2,0,0) × 2/*a* positions, expressed by the formula type 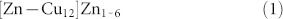
This formula (1) describes the Cu-Zn alloys showing relatively strong short-range-order tendencies in both the 1^st^ and the 2^nd^ neighbors, covering a composition range of [Zn-Cu_12_]Zn_1_ and [Zn-Cu_12_]Zn_6_, or 14.3 ≤ at.% Zn ≤ 36.8, 14.6 ≤ wt.% Zn ≤ 37.5.
3. In accordance with the weaker Warren-Cowley short-range-order parameters in the Zn-lean alloys^13^, the six 2^nd^-neighbors at the (2,0,0) × 2/*a* positions are occupied by a mixture of Cu and Zn, expressed by the formula type 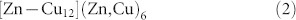
This formula (2) describes the Cu-Zn alloys showing relatively weak short-range-order tendencies in the 2^nd^ neighbors, covering a composition range of [Zn-Cu_12_]Cu_6_ and [Zn-Cu_12_](Zn_1_Cu_5_), or 5.3 ≤ at.% Zn ≤ 10.5, 5.4 ≤ wt.% Zn ≤ 10.8.

Therefore, in accordance with the formula types (1) and (2), stable Cu-Zn solid solutions exist within a composition range of 5.3 ≤ at.% Zn ≤ 36.8, or 5.4 ≤ wt.% Zn ≤ 37.5.

In real solid-solution alloys, different degrees of disordering should be present, and mixed atomic occupancies should occur. For instance, in accordance with the Warren-Cowley short-range-order parameters, *α_n_*, measured in a single crystal Cu_68.9_Zn_31.1_ (the subscript numbers after the elements indicate atomic percents or atomic fractions) alloy^12^, the Zn-centered 1^st^- and 2^nd^-neighbor shells consist, respectively, of Cu_9.4_Zn_2.6_ and Cu_3.5_Zn_2.5_. The chemical composition within the 2^nd^-neighbor local zone is then Zn + Cu_9.4_Zn_2.6_ + Cu_3.5_Zn_2.5_ = Cu_12.9_Zn_6.1_, or Cu_67.9_Zn_32.1_ in at.%, which is close to that of the alloy, Cu_68.9_Zn_31.1_. By alternating Zn in the 1^st^ shell with Cu in the 2^nd^ shell until the twelve 1^st^-neighbor sites are completely occupied by Cu, an idealized cluster formula is then reached [Zn-Cu_12_](Cu_0.9_Zn_5.1_).

To satisfy the ideal atomic interactions between Cu and Zn, i.e., the 1^st^-neighbor shell fully occupied by Cu and the 2^nd^-neighbor sites by Zn, the Cu atoms in the 2^nd^-neighbor sites are removed (they become 1^st^ neighbors of nearby [Zn-Cu_12_] clusters). Thus, the glue atoms now consist purely of Zn. The closest integer form of this formula is then [Zn-Cu_12_]Zn_5_ after nearly one Cu atom is removed, which corresponds to the composition of the specification C27000 (65Cu-35Zn, yellow brass). The idealized cluster formulas then give the averaged pictures at the 1^st^ and 2^nd^ neighbors. Structures described by such cluster formulas should possess relatively high structural stabilities, because atoms are so arranged in the neighborhood configurations that their atomic interactions are best respected. For this reason, we here intend to term the solid solutions possessing such ideal short-range orders as the ‘stable solid solutions’.

## Cu-Zn Brass Composition Interpretation

In the following, the compositions of the Cu-Zn α-brass industrial alloys from the American Society for Testing Materials (ASTM) standards^9^ will be checked, using the proposed cluster formulas of types (1) and (2), as listed in Table 1.

The two Zn-lean alloys, C21000 (95Cu-5Zn) and C22000 (90Cu-10Zn), would be formulated according to the formula type (2) into [Zn-Cu_12_]Cu_6_ (94.6Cu-5.4Zn) and [Zn-Cu_12_]Cu_5_Zn_1_ (89.2Cu-10.8Zn), respectively.

The alloys with more Zn contents fit the formula type (1). C23000 (85Cu-15Zn), C24000 (80Cu-20Zn), C26000 (70Cu-30Zn), C27000 (65Cu-35Zn, previously C26800 with 66Cu-34Zn), and C27400 (63Cu-37Zn) would be formulated by type (1), [Zn-Cu_12_]Zn_1,2,4,5,6_, the last composition corresponding nearly to the solubility limit of Zn in α-brass at room temperature. The formulated compositions deviate from the specified ones by less than 1 wt.%.

The missing formula, [Zn-Cu_12_]Zn_3_ (74.5Cu-25.5Zn), does not correspond to any specification, apparently due to easy ordering of the Cu_3_Zn type near this composition.

C22600 (87.5Cu-12.5Zn) and C28000 (60Cu-40Zn) cannot be explained. The former one does not show special mechanical properties but is used for its golden color. The latter alloy, known as Muntz alloy, is actually dual-phased (precipitation of β-CuZn) and the proposed formulas, destined to a single-phase state, would fail.

More industrial alloys are being analyzed by us to check the universality of the cluster-formula approach in understanding the alloy selections. Here we show the Cu-Ni industrial alloys as the typical example for single-phase FCC solid-solution alloys with a weak positive enthalpy of mixing (*ΔH*_Cu-Ni_ = +2 KJ/mol, in comparison with *ΔH*_Cu-Zn_ = −6 KJ/mol). Coincidently, the Warren-Cowley short-range-order parameters in this system are quite small, with α_1_ = 0.058 and α_2_ = −0.058 for the Cu_80_Ni_20_ alloy^14^. It was also pointed out^15^ that in binary Cu-Ni solid solutions, the Cu-Cu nearest-neighbor short-range order dominates, and there exist [Cu-Cu_12_] clusters, which are irrelevant to composition variations. Then the formula similar to the formula type (2), [Cu-Cu_12_](Cu,Ni)_6_, should be adopted in the explanation of the Cu-rich Cu-Ni alloy compositions. The Cu-rich specifications, C70400 (95Cu-5Ni), C70600 (90Cu-10Ni), C70900 (85Cu-15Ni), C71000 (80Cu-20Ni), C71300 (75Cu-25Ni), and C71500 (70Cu-30Ni), are respectively explained with [Cu-Cu_12_](Cu_5_Ni_1_) (95.1Cu-4.9Ni), [Cu-Cu_12_]Cu_4_Ni_2_ (90.2Cu-9.8Ni), [Cu-Cu_12_](Cu_3_Ni_3_) (85.2Cu-14.8Ni), [Cu-Cu_12_]Cu_2_Ni_4_ (80.2Cu-19.8Ni), [Cu-Cu_12_](Cu_1_Ni_5_) (75.2Cu-24.8Ni), and [Cu-Cu_12_]Ni_6_ (70.1Cu-29.9Ni).

On the Ni-rich side, the cluster should be altered to [Ni-Ni_12_], which is then glued with six Cu and Ni atoms following the formula type (2). The composition of the only known Ni-rich alloy, as represented by Monel 400 specified by 28.0 ~ 34.0 wt.% Cu, is bounded by two formulas, [Ni-Ni_12_]Cu_5_Ni (27.9Cu-72.1Ni) and [Ni-Ni_12_]Cu_6_ (33.3Cu-66.7Ni), again of the formula type (2).

The revelation of the composition formulas for FCC-type industrial alloys, as exemplified by Cu-(Zn,Ni) alloys here, and together with what proposed previously for Fe-containing Cu-Ni alloys, [Fe-Ni_12_]Cu_x_^3^, maraging stainless steels, [Ni-Fe_12_](Cr_2_M_1_), M being alloying elements^4^, and β-Ti bio-alloys, [Mo_0.5_Sn_0.5_-Ti_14_]Nb^5^, points to simple composition rules in terms of cluster formulas for all kinds of industrial alloys. The composition interpretation is much simplified, because the cluster formulas describing short-range-order structural units involve a dozen of atoms only. New alloys can be developed by substitutions in the basic formulas, thus opening up a fundamentally new route towards alloy design.

## Author Contributions

H.L.H. collected the composition and property data. Q.W. analyzed the compositions. C.D. proposed the model. P.L. helped with composition interpretation. All authors participated in writing the paper.

## Figures and Tables

**Table 1:** Typical Cu-Zn α-brass industrial alloys in ASTM specifications^9^ and their composition interpretations in terms of the cluster formulas

Specifications (wt.%)	Names	Cluster formula (wt.%)	Type
C21000 (95Cu-5Zn)	Gilding metal	[Zn-Cu_12_]Cu_6_ (94.6Cu-5.4Zn)	(2)
C22000 (90Cu-10Zn)	Commercial bronze	[Zn-Cu_12_]Cu_5_Zn_1_ (89.2Cu-10.8Zn)	
C22600 (87.5Cu-12.5Zn)	Jewelry bronze	-	
C23000 (85Cu-15Zn)	Red brass	[Zn_1_-Cu_12_]Zn_1_ (85.4Cu-14.6Zn)	(1)
C24000 (80Cu-20Zn)	Low brass	[Zn-Cu_12_]Zn_2_ (79.6Cu-20.4Zn)	
Cu_3_Zn	-	[Zn-Cu_12_]Zn_3_ (Cu74.5-25.5Zn)	
C26000 (70Cu-30Zn)	Cartridge brass	[Zn-Cu_12_]Zn_4_ (Cu70.0-70.0Zn)	
C26800 (65Cu-35Zn, previously 66Cu-34Zn)	Yellow brass	[Zn-Cu_12_]Zn_5_ (66.0Cu-34.0Zn)	
C27000 (65Cu-35Zn)	Yellow brass	[Zn-Cu_12_]Zn_5_ (66.0Cu-34.0Zn)	
C27400 (63Cu-37Zn)	Common brass	[Zn-Cu_12_]Zn_6_ (62.5Cu-37.5Zn)	
C28000 (60Cu-40Zn)	Muntz metal	-	
